# Mucocele: a rare complication following stapled haemorrhoidopexy

**DOI:** 10.1186/s12893-022-01744-3

**Published:** 2022-08-01

**Authors:** Xing-Yang Wan, Yuan-Ji Fu, Gui-Ming Li, Guo-Zhong Xiao, Zhi-Wei Guo, Dong-Lin Ren, Bo Cao, Hong-Cheng Lin

**Affiliations:** 1 Department of Coloproctology Surgery, The Sixth Affiliated Hospital of Sun Yat-Sen University (Gastrointestinal and Anal Hospital of Sun Yat-Sen University), 26 Yuancun Erheng Road, Heng Lu, Guangzhou, 510655 China; 2 Guangdong Provincial Key Laboratory of Colorectal and Pelvic Floor Diseases, Guangzhou, China; 3 Guangdong Institute of Gastroenterology, Guangzhou, China; 4 Department of Anorectal, The Eighth Hospital of Wuhan, Wuhan, 430000 China; 5 Department of Colorectal and Anal Surgery, The First Affiliated Hospital of GuiZhou College of Traditional Chinese Medicine, Guiyang, 550001 China

**Keywords:** Mucocele, Stapled haemorrhoidopexy, Complication

## Abstract

**Background:**

Stapled haemorrhoidopexy (SH) has resulted in a unique collection of procedural complications with postoperative mucocele a particularly rare example. This study is designed to comprehensively describe the characteristics of rectal mucocele and discuss its pathogenesis following SH surgery.

**Methods:**

A database of patients presenting with a rectal mucocele following an SH procedure was established and studied retrospectively.

**Results:**

Seven patients (5 males; median age 32 years, range 20–75 years) were identified. All patients complained of variable anal discomfort with 5/7 presenting with inconstant anal pain, 2 with de novo evacuatory difficulty. These cases appeared at a median time of 6 months (range 2–84 months) after SH surgery.

**Conclusion:**

Rectal Mucocele develops when mucosal fragments become embedded and isolated under the mucosa. It is a preventable complication of SH surgery by ensuring correct purse string placement prior to stapled haemorrhoid excision.

**Supplementary Information:**

The online version contains supplementary material available at 10.1186/s12893-022-01744-3.

## Background

Haemorrhoids are the commonest anorectal disorder where it is estimated that 50% of the population over the age of 50 years suffer from the condition [1]. Traditional open haemorrhoidectomy remains the gold standard in the surgical management of circumferential prolapsed haemorrhoids [2], moreover, many surgeons are attempting to use stapled heamorrhoidopexy (SH) because of reported advantages over open variants of hameorrhoidectomy which include a shorter operating time, less postoperative pain, better wound healing and an earlier return to work [3, 4]. In general, SH increased relative risk of haemorrhoid recurrence compared with traditional open haemorrhoidectomy [5, 6]. The stapled approach to the haemorrhoid-bearing mucosa has resulted in a unique collection of procedural complications [7–11] with postoperative rectal mucocele (RM) a particularly rare example [12]. The clinical presentation by patients with a RM is typically delayed with some asymptomatic cases diagnosed as an incidental finding. Symptoms of a post-SH RM may include tenesmus and rectal discomfort within some cases an exacerbation of a pre-existing evacuatory difficulty. We suppose it might be caused by a distinct technical consequence of the stapled technology resulted from incorrect placement of the resected rectal mucosa under the stapled line. In this circumstance, part of the rectal mucosa is either not incorporated into the suture or has subsequently pulled away from the purse-string [13]. The ultimate result is that the epithelium embedded submucosally either with or without a luminal connection [14, 15]. We report a series of cases of symptomatic RM following an SH procedure. Therefore, the retrospective study aims to access the possible causes of RM through clinical characteristics, management, and outcomes of this rare complication.

## Patients and methods

Ethical permission for conduct of this retrospective study was obtained from the Ethics Committees of the Sixth Affiliated Hospital of Sun Yat-sen University. Data since 2013 were collected from a hospital database with identification of cases with a RM following an SH procedure (Additional file 1: Table S1). The patients with incomplete medical history data are excluded. All SH procedure were performed the following described SH technique [16]: Anal dilatation was done at the beginning of the procedure. After full dilatation, at the upper end of the dentate line of approximately 3–4 cm, patients were sutured with a purse in the rectal submucosa. For the cases with large prolapsing hemorrhoids, double purse string was recommended. The stapler was opened to its maximum position and positioned proximal to the purse string. The purse string was then tightened. After tightening the purse line, the purse line was effectively drawn out at the side of the stapler, and the stapler was tightened to keep the stapler closed for 20–30 s to ensure hemostasis. Generally, stapled line would be checked and suture if necessary. Analysis included 7 patients managed at the Sixth Affiliated Hospital of Sun Yat-sen University. All the patients were diagnosed according to the previous operation history, clinical symptoms, and radiological assessment such as MRI or CT. Digital rectal examination revealed a pararectal mass in all cases. Specialized radiology was utilized to rule out differential diagnosis including benign neoplasms (lipoma, leiomyoma, GIST), malignant tumours, (mesenchymal, neural, neuroendocrine, carcinoid, lymphoma) endometriotic deposits, cystic developmental retrorectal lesions (rectal duplication, epidermoid and dermoid cysts) and rectal diverticula. MRI was performed with five patients while five with colonoscopy, and only three patients underwent endosonography. Demographic and clinicopathological data were stored on a secured database with recording of gender, age, the location of the pararectal mass, details of the primary surgical and subsequent surgical procedures, pathological characteristics, and postoperative symptoms.

The management varied with en bloc excision in 2 cases and incision in the remaining 5 patients. In this latter group, the cyst was laid open with a wide marsupialization and the mucocele wall was cauterized with instillation of dehydrated alcohol or electric knife in the largest two cysts. Visible residual staples were removed. The mucoid contents were sent for cytological examination and a biopsy of the pocket wall was sent for histology. Patients were routinely followed-up with recurrence determined clinically and symptomatically.

### Statistical analyses

Categorical variables are numerically stated with continuous variables presented as medians (and ranges).

## Results

Seven patients (5 males; median age 32 years, range 20–75 years) were identified with a RM following an SH procedure since 2013. Table 1 summarizes the demographic data and clinical information of all patients. These cases appeared at a median time of 6 months (range 2–84 months) after SH surgery. One of the patients had a comorbid illness (Type II diabetes mellitus). All patients complained of variable anal discomfort with 5/7 presenting with inconstant anal pain, 2 with defecate difficulty. None of the patient presented with intermittent fevers and rectal bleeding. Digital rectal examination revealed pararectal mass in every case which was anterior in 1, anterolateral in 3, posterolateral in 2, lateral in 2 and posterior in 1, two masses were palpated in two patients. These masses were morphologically heterogeneous comprised mostly of cystic components with a variable size ranging from 0.5 × 0.8 cm up to 3.3 × 4.5 cm.

**Table 1:** Demographic and clinical characteristics of the patients

Variable	No. of patients (n = 7)
Sex: male/female	5/2
Age (years)	32 (20–75)
Time after PPH surgery (months)	6 (2–84)
Symptoms	
Evacuatory difficulty	2
Anal pain	5
Fever	0
Rectal bleeding	0
Primary surgical procedure	
PPH	7
The second surgical procedure	
En bloc cyst excision	2
Incision	5
Pararectal mass location	
Anterior	1
Anterolateral	3
Posterolateral	2
Lateral	2
Posterior	1
Pathological features	
Inflammatory granulation tissue	4
Fibrocystic tissue	2
Necrotic material	1

**Fig. 1:**
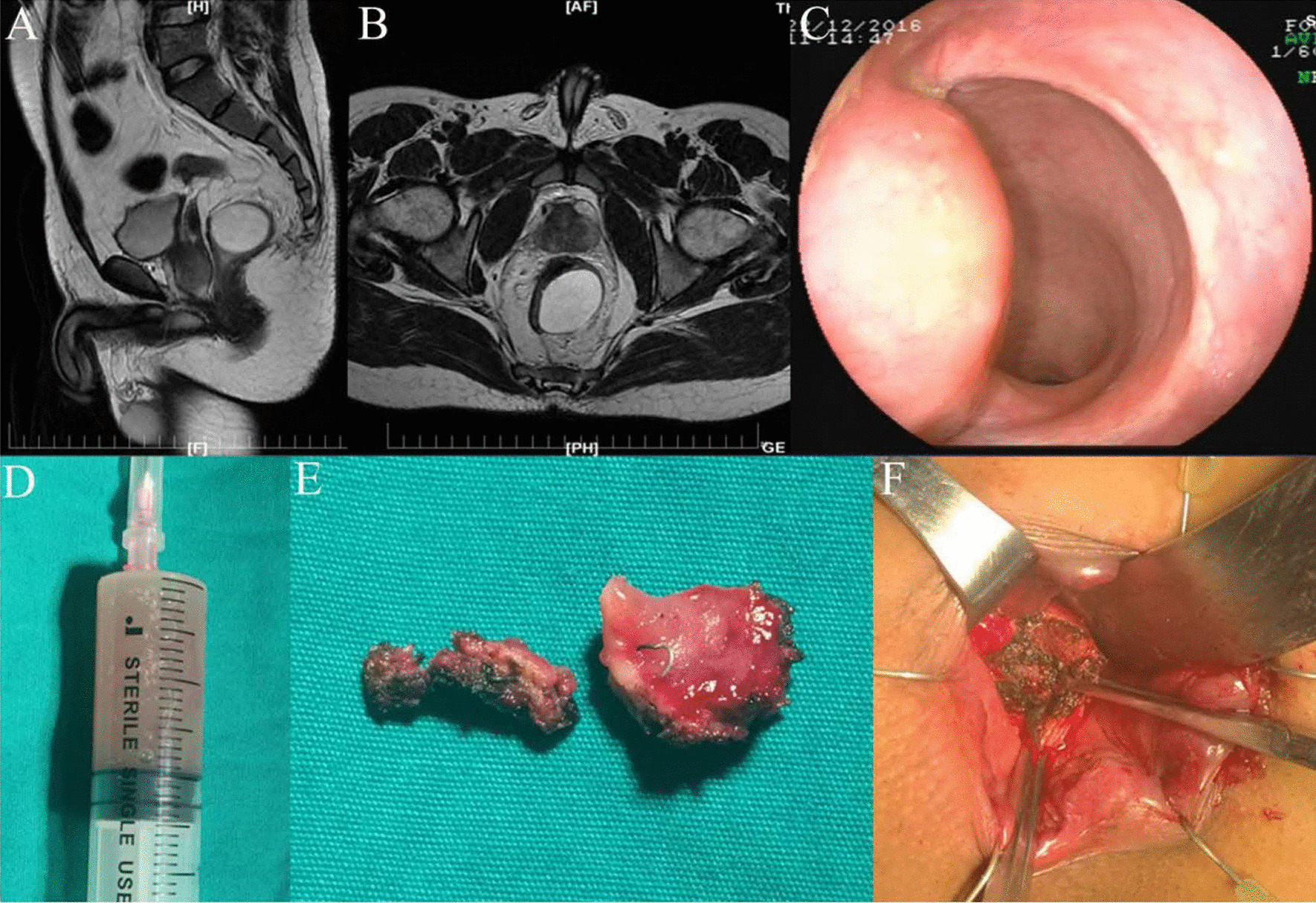
MRI, colonoscopic and operative findings of the lateral pararectal mass in the same patient.** A** Sagittal image showing a large mucocele without sphincteric involvement (**A**). Image **B** confirms the mucocele locale and dimensions on axial MRI. **B** On flexible endoscopy the mucocele appears as a pararectal submucosal mass without any luminal connexion. **C** Clear mucoid material was evacuated from the mass. **D** The operative specimen included part of the rectal mucocele with visible rectal mucosa and retained staples. **F** Operative view in which the mucocele was formally opened and marsupialized with the rectal lumen

In each case the mass was closely associated with visible residual staples even when there was no luminal connection. The cytology of the mucus was unremarkable. Histology showed normal rectal mucosa in each case with only some surrounding fibrosis. Figure 1 shows an MRI along with the findings on colonoscopy and operative appearance of the pararectal mass in the same patient which was located laterally between the 2 o’clock and 5 o’clock positions in lithotomy with a size of 3.3 × 4.5 cm. In this case, the mass extended above the levator floor, the mass was performed with the incision procedure. Only two patients were assessed with repeat MRI examination one week after surgery and the postoperative course of all patients was unremarkable with all cases asymptomatic at a one-year follow-up.

## Discussion

The procedure for prolapse and hemorrhoids (PPH), the first kind of SH, was introduced in 1998 by Longo [17] and is recommended for patients with Grade II–IV haemorrhoids [18]. RM following SH procedure is extremely uncommon representing about 2.5% of complications [7]. RM is particularly rare in situations where there has not been a prior stapled anorectal procedure (SH or STARR transanal rectal resection) [19–21].

**Fig. 2:**
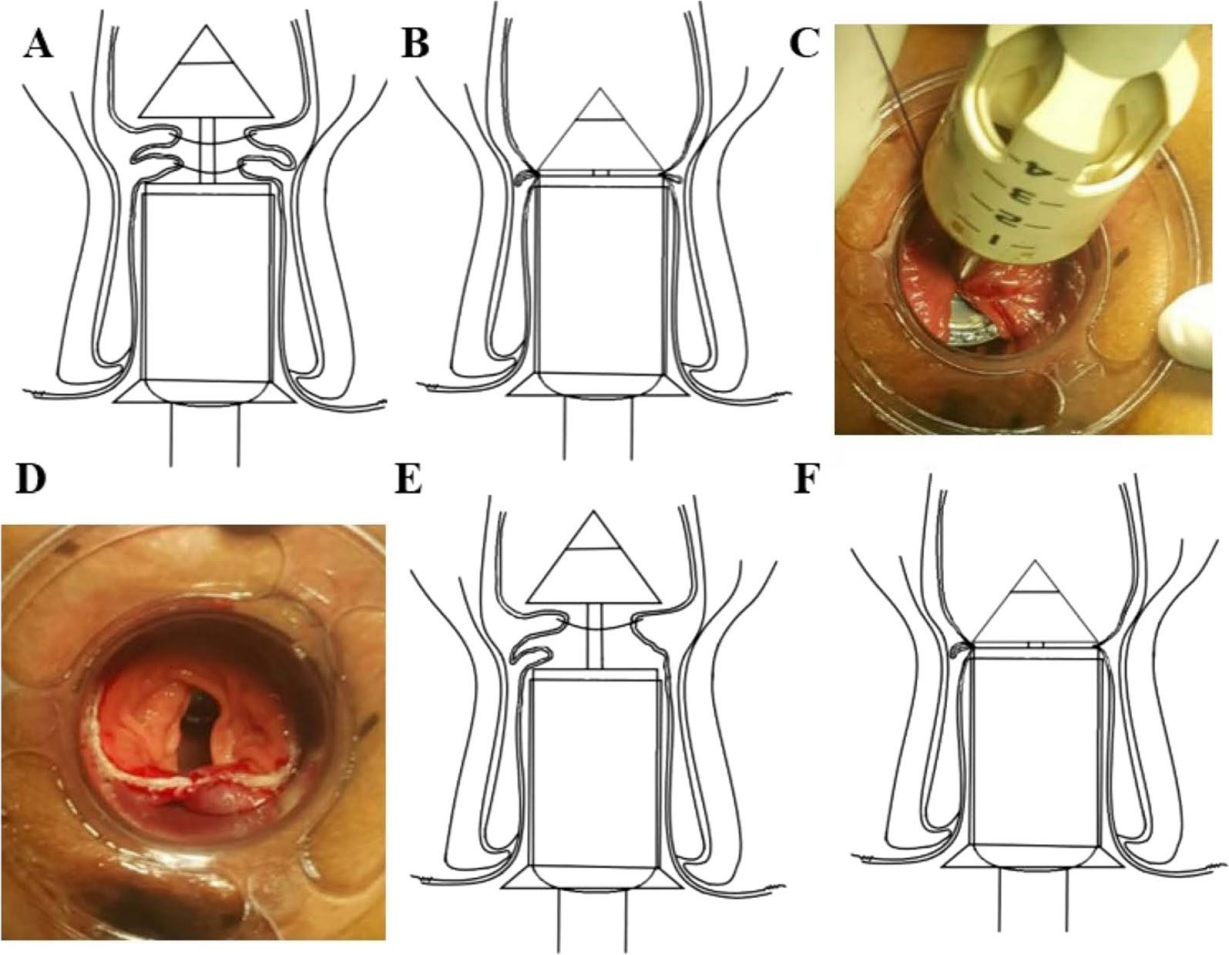
Proposed mechanisms of mucocele formation after SH—double purse string and single purse string approaches. **A** Open configuration of the stapler with a double purse string. **B** Configuration after stapler firing. When a double purse string is used widely placed sutures can lead to a small rectal mucocele that is excluded from the stapled cavity during the stapler firing. **C** A PSH (partial stapled hemorrhoidopexy) stapler prior to firing. **D** After firing unresected tissue forms a mucosal bridge. **E** With a single purse string a loose prolapsing fold of rectal mucosa can be excluded with separation after stapler firing (**F**)

The association of RM with an SH procedure represents a specific variant of the rectal pocket syndrome originally described by Pescatori et al. [22] where secretory fragments of rectal mucosa become isolated and embedded into the submucosa. With rectal pocket syndrome there is a luminal connexion which permits the accumulation and concretion of a faecolith and its attendant potential complications. By contrast, with a postoperative RM, there is no direct luminal connexion resulting in the build-up of mucus and the formation of a pararectal mass. Figure 2 is a schematic representation of the suggested mechanisms for the pathogenesis of a RM following an SH procedure. The goal of SH is to incorporate all the mucosal folds so that the mucosa is evenly pulled up as the gun is closed. Generally, for either a rectal pocket or a RM to develop, there needs to be a separation of part of the mucosal margin after stapled excision and anastomosis so that the mucosa retained under the stapled line. These different suggested mechanisms of formation of a RM are then dependent upon the positioning of a purse string and the distance between double-purse string sutures. Summarizing previous research conclusions, there are three mechanisms leading to the formation of RM. Firstly, when a double purse string suture is employed, some of the tissue between widely placed sutures may not be completely retracted into the stapler cabin housing leading to closed cavities being formed once the stapler is fired (Fig. 2A, B). Secondly, as the purse string suture is tightened and tied around the shaft of the opened stapler head, inadvertent incorporation of extra mucosa between the stapler jaws leading to the formation RM (Fig. 2E, F). Thirdly, when single purse-string was performed, the distance between two stiches is too excessive or slippage of the purse-string result in the bite of tissue is too superficial can result in RM. However, we do not consider that RM will be result when the third condition occurs alone. In general, it seems purse string suture where there is an excessive distance between the stitches can leave out part of the circumferential mucosal margin. And a purse string suture which has been placed too superficially may pull through leaving a component of the mucosa that is not incorporated into the anastomosis when the stapler has been fired. Our group had first reported another safety and feasibility of a modified SH procedure called tissue-selecting technique, which is a partial or segmental stapled hemorrhoidopexy [23]. It is also referred to as partial stapled hemorrhoidopexy (PSH). Actually, with PSH stapler (where a single purse string is used) only a mucosal bridge, not a RM or rectal pocket syndrome, is created, when the sutures are placed either too widely or are too superficial. In this circumstance there is no opportunity to form a closed space cavity. Because the stapler-forming anastomotic loop is linear and perpendicular to the rectal wall, and there is no anastomotic line is formed parallel to the rectal wall. In other words, only too excessive or superficial suture is not enough to cause RM formation. In addition to stapler-related reasons, cyst formation may also be caused by improper surgeon's manipulation. In some cases, the patient may have silk sutures for hemostasis during or after surgery. We supposed that this may also resulted in the embedding of islands of mucosa and was exacerbated by the use of using a nonabsorbable suture. These different suggested mechanisms of formation of a RM are then dependent upon the distance between sutures and the loose rectal mucosa which could not be pulled into the stapled cavity completely. As the mucosa was folded without no opening direct connection with rectal lumen, and mucus could be secreted secretory mucosa constantly to form cysts. Finally, abscesses can be formed due to residual feces or bacteria exacerbating the infection.

Although the majority of such mucoceles are thought to be asymptomatic, once the rectal epithelium is embedded submucosally there will be a cycle of mucus retention, faecal contamination, and bacterial overgrowth with the potential for abscess (and even fistula) formation. Symptomatic cases outside of a septic presentation will typically complain of tenesmus and even incomplete evacuation, where the presence of a pararectal mass on clinical examination (or with specialized radiology) has its own differential diagnosis [24]. This may include benign neoplasms (lipoma, leiomyoma, GIST), malignant tumours, (mesenchymal, neural, neuroendocrine, carcinoid, lymphoma) endometriotic deposits, cystic developmental retrorectal lesions (rectal duplication, epidermoid and dermoid cysts) and rectal diverticula [14, 25, 26]. The use of MR imaging defines the characteristics of the contents of any pararectal mass as well as its connexion to the central rectal lumen. Cystic masses are characterized by high signal intensity on T2-weighted sequences with inflammatory changes evident in those where there is entrapped faecal residue and surrounding oedema of the cyst wall [24].

When indicated for symptoms, surgery for RM is the treatment of choice [12]. The surgical options may include mucocele excision or often more simply, evacuation of the mucocele contents, creation of an opening of the cyst into the rectal lumen (marsupialization) with removal of extraneous staples. The choice of surgical depends on the location and size of the mucocele, and smaller mucocele located in the superficial area can often choice mucocele excision. Na et al. [25] reported their results of transanal diverticulectomy with direct repair of the rectal wall in mucocele cases after PPH procedures. The surgery varies depending upon the location and size of the mucocele with surgical combinations used when required.

According to our experience, the incidence of RM can be reduced when standard stapled haemorrhoidopexy is performed with some tricks. We believe that purse suture errors still are the main cause of RM. We mainly pay attention to the skill of purse suturing. If double purse-string is performed, the distance between the two rows of purses should not be too far. Before the staple cartridge is completely closed, the purse-string traction line should be continuously tightened so that the sutured mucosa enters the staple cartridge evenly. The closed stapler should be fully squeezed for 20–30 s to reduce the formation of hematoma and reduce postoperative suture hemostasis. In addition, emphasis on adequate disinfection process can reduce the risk of bacterial retention during suturing. In addition, more attention should be paid to the integrity of specimen inspection to detect early problems of anastomosis.

The limitation of this study is its retrospective nature. Moreover, RM is a rare complication, we hope to collect more cases to support the theory we proposed for the mechanisms RM after SH procedure.

## Conclusion

We propose several mechanisms that can lead to RM formation after an SH procedure whereby mucosal fragments become isolated and embedded submucosally. Although these rather rare mucoceles can be managed relatively effectively by deflation and fenestration into the rectal lumen, they are preventable with some surgical tricks.

## Supplementary Information

**Additional file 1**: **Table S1.** The database of patients presenting with a rectal mucocele following an SH procedure.

## Data Availability

The datasets of the current study have been provided in Additional files.
